# Age-, Sex- and Region-Specific Patterns in Sensitization Rates to Food Allergens and Food Allergy Prevalence in Croatian Children: The H2020 IMPTOX and ERDF P4 Study Findings

**DOI:** 10.3390/children13020234

**Published:** 2026-02-06

**Authors:** Jan Pantlik, Marcel Lipej, Ivana Banić, Maja Šutić, Sandra Mijač, Petra Anić, Ana-Marija Genc, Ana Vukić, Antonija Piškor, Adrijana Miletić Gospić, Željka Vlašić Lončarić, Milan Jurić, Vlatka Drinković, Ivana Marić, Tin Kušan, Mirjana Turkalj

**Affiliations:** 1 Department of Medical Research, Srebrnjak Children’s Hospital, HR-10000 Zagreb, Croatia; 2 IT Department, Srebrnjak Children’s Hospital, HR-10000 Zagreb, Croatia; 3 Department of Innovative Diagnostics, Srebrnjak Children’s Hospital, HR-10000 Zagreb, Croatia; 4 Department of Allergy and Clinical Immunology, Srebrnjak Children’s Hospital, HR-10000 Zagreb, Croatia; 5 Department of Pulmonology, Srebrnjak Children’s Hospital, HR-10000 Zagreb, Croatia; 6 Faculty of Medicine, J.J. Strossmayer University of Osijek, HR-31000 Osijek, Croatia; 7 Faculty of Medicine, Catholic University of Croatia, HR-10000 Zagreb, Croatia

**Keywords:** allergic sensitization, food allergy, polysensitization, sensitization patterns, children

## Abstract

**Background/Objectives:** Food allergy (FA) is a substantial health burden in children. FA is often associated with malnutrition and malabsorption, due to restrictive food avoidance diets, which can significantly impair the patient’s and their family’s quality of life. To this date, population-based data combining sensitization and clinical allergy remain limited. This study aimed to assess the patterns of sensitization rates to food and food allergy prevalence rates in Croatian children and to evaluate differences according to age, sex, and region of origin. **Materials and Methods:** In this cross-sectional study, 1948 preschool and school-aged children from three Croatian regions (Zagreb, Dalmatia, and Slavonia) were included. Participants underwent skin prick testing to common food and inhalant allergens. Data on personal and family medical history were collected using questionnaires and medical records. FA prevalence was evaluated using self-reported data in school-aged children and physician-diagnosed FA data in preschool children. **Results:** Overall, 41% of participants were sensitized to at least one allergen, while 13% were sensitized to at least one food allergen. Tree nuts—particularly hazelnut—were the most common food-derived sensitizers, followed by hen’s egg, cow’s milk, and fish. Boys exhibited higher total sensitization rates than girls (44.2% vs. 37.5%; *p* = 0.001), higher food allergen sensitization rates (14.7% vs. 11.4%; *p* = 0.037), and higher total polysensitization rates (30.7% vs. 22.6%; *p* < 0.001). School-aged children showed higher total sensitization (44.8% vs. 33.4%; *p* < 0.001) and polysensitization rates (29.8% vs. 20.5%; *p* < 0.001) than preschool children, while sensitization to food allergens did not differ between age groups. Food allergen sensitization rates differed by region, with higher prevalence in Zagreb compared with Dalmatia and Slavonia (*p* = 0.0055), whereas total sensitization rates did not differ regionally. The agreement between sensitization and self-reported FA among school-aged children was low (κ = 0.22; *p* < 0.001), as was the agreement between sensitization and physician-diagnosed FA in preschool children (κ = 0.13; *p* < 0.001), despite high specificity in both analyses (95% and 99%%, respectively). **Conclusions:** Allergic sensitization is common among Croatian children, but it poorly predicts clinically relevant food allergy. These findings highlight the multifactorial nature of allergen sensitization in children and emphasize the need for improvements in diagnostic pathways, targeted prevention strategies, and continued surveillance to optimize allergy prevention and management in children.

## 1. Introduction

Food allergy (FA) is a growing global health issue, particularly in the pediatric population. According to certain studies, more than 50% of patients with food allergies are not adequately diagnosed and/or treated [1]. Food allergies are long-term, chronic and often progressive diseases that require complex and ongoing healthcare, and the costs of their treatment are constantly increasing. Experts estimate that the indirect costs of insufficiently treated allergies, including FA, are in the EU range from 55 to 155 billion EUR annually [2]. Most treatment and prevention options in FA are based on food avoidance diets, which are often restrictive and may lead to malnutrition and malabsorption in children. Additionally, food allergies significantly impair the patient’s quality of life, bring certain stigmas and psychological issues due to the inability to consume certain foods, and are a major socio-economic burden for the individual and their families, due to the increased costs of food declared allergen-free [3].

FA prevalence has been on the rise in the past few decades, reflecting changes in environmental factors, dietary habits, and possibly early-life microbial exposures [4,5]. Allergic reactions to food range from mild symptoms, such as local skin and gastrointestinal issues (irritation and discomfort), to severe, life-threatening anaphylactic reactions. The rising prevalence of these conditions poses a considerable burden on families and healthcare systems, particularly in pediatric populations [6]. A number of hypotheses attempt to explain the increasing prevalence of FA. The hygiene hypothesis suggests that reduced microbial exposures in early life, due to improved sanitation and decreased exposure to farm environments, may contribute to an imbalance in immune system development, favoring allergic responses [7]. Another theory highlights the role of dietary changes, such as reduced consumption of fresh produce and increased intake of processed foods, in altering gut microbiota and immune responses [8]. Additionally, environmental pollution and climate change likely influence allergenicity and immune system interactions, although these mechanisms are not yet fully understood [9].

FA prevalence varies globally and is affected by factors such as genetics, cultural dietary practices, and environmental exposures. In Western countries, studies report food allergy prevalence rates of up to 10% in children, with peanuts, milk, and shellfish being among the most common allergens [9,10]. In contrast, developing regions may report lower prevalence rates, although underdiagnosis remains a challenge due to limited access to diagnostic tools and awareness [11]. Despite extensive research in high-income countries, data from Southeastern Europe, including Croatia, are limited, leaving significant gaps in understanding the regional patterns of food allergen sensitization. One study involving preschool children in Croatia (up to the age of 4 years) reported an FA prevalence rate of 5.4% [12].

In large epidemiologic studies, skin prick testing (SPT) is a commonly used diagnostic method to identify allergic sensitization, a key prerequisite in IgE-mediated hypersensitivity reactions. However, a positive SPT result indicates sensitization but not necessarily clinically relevant allergy, as not all sensitized individuals experience allergic reactions [13]. The combination of SPT with detailed clinical questionnaires, as well as self-reporting allergic conditions, can provide a more comprehensive picture of food allergy prevalence and patterns [14]. Understanding the relationship between sensitization and clinically significant allergies is essential for designing effective public health strategies and clinical interventions. Additionally, polysensitization and allergy to multiple food allergens are known to increase the risk for additional sensitizations and allergic multimorbidity [15].

In Croatia, children’s dietary patterns are influenced by cultural and regional differences. For instance, Mediterranean diets rich in fish and nuts are prevalent in coastal areas, while diets in continental regions may emphasize dairy and grains [16]. These dietary habits may contribute to distinct sensitization patterns, reflecting regional differences in allergenic exposures. However, systematic studies examining these regional variations in food allergen sensitization and their clinical implications are scarce.

This study aims to address the lack of comprehensive data on food allergen sensitization in the pediatric population in Croatia. By analyzing data from 1948 children aged 1 to 18 years across three distinct regions—the Zagreb region, Slavonia region, and Dalmatia region—we seek to identify sensitization rates to 14 food allergens using SPT. This study also incorporates self-reported food allergy data obtained through detailed questionnaires, enabling a comparison between sensitization and clinically relevant allergies. To the best of our knowledge, this study is the first one investigating the sensitization and FA profiles in Croatian children, aimed at understanding region-, age-, and sex-specific patterns and their determinants, which may help develop targeted interventions, such as dietary recommendations and educational campaigns, to mitigate the impact of FA on patients’ and their families/quality of life.

## 2. Materials and Methods

These single-center (lead by the Srebrnjak Children’s Hospital in Zagreb, Croatia), cross-sectional, longitudinal and observational cohort studies were stratified by age, sex and residential area and involved pediatric participants aged 1–6.5 and 6.5–18 years, based on differential levels of environmental exposure and susceptibility to allergic diseases (allergic vs. healthy non-allergic participants, preschool and schoolchildren), as part of 2 research projects: Horizon 2020 IMPTOX (an innovative analytical platform to investigate the effect and toxicity of micro- and nanoplastics combined with environmental contaminants on the risk of allergic disease in preclinical and clinical studies; grant agreement number: 965173) and ERDF P4 [17].

Participants were recruited from three distinct geographical regions in Croatia: 2 continental regions (Zagreb, the capital city, which is predominantly urban) and Slavonia (mixed rural and urban) and the Mediterranean region of Dalmatia (also mixed rural and urban), which differ in the levels of environmental exposure (to pollutants and allergens) as well as certain lifestyle and dietary habits.

### 2.1. Study Population

Participants were recruited in schools (children aged 6.5–18 years, in elementary schools and high schools) and preschool institutions (children aged 1–7 years) in 3 distinct geographical regions in Croatia, differing in a number of environmental and lifestyle factors, including dietary habits. The age span in each of the cohorts reflects the educational system in Croatia, where children start elementary school at the age of 6.5 years at the earliest, while some, due to their birth date, start elementary school at the age of 7 or 5.5 years.

Written informed consent was obtained from the children’s parents/caregivers. The study protocols were approved by the Ethics Committee at the Srebrnjak Children’s Hospital on 19 October 2021 (the H2020 IMPTOX study, CLASS: 100-02/21-01, Rec. No.: 04-930/3-21, NCT05177744), and on 14 March 2022 (the P4 study, CLASS: 100-02/22-01, Rec. No.: 04-300/1-22, NCT05462444).

In the period of October 2022 to April 2024, a total of 1155 participants, aged 6.5–18 years in the IMPTOX study and a total of 793 participants aged 1–7 years in the P4 study were recruited. The participants were children of both sexes, whose parents/caregivers agreed for them to participate in the studies after receiving both oral and written information about this study to their satisfaction, and those not meeting the criteria for exclusion. The criteria for inclusion and exclusion were assessed by a specialist physician (a pediatric allergy specialist with 20+ years of expertise in the field). The criteria for inclusion were children at the ages of 1–7 years (P4) and 6.5–18 years (IMPTOX), including both healthy and non-allergic as well as allergic participants. Additional inclusion criteria included previously confirmed sensitization to at least one food allergen or inhaled allergens, and/or having a diagnosis of at least one allergic disease (allergic rhinitis, allergic asthma, atopic dermatitis, and/or food allergy) for at least one year. The exclusion criteria were known malignancies and congenital and other serious chronic illnesses preventing reliable results (such as systemic mastocytosis, severe metabolic disorders, etc.). Furthermore, children were excluded from participation and collection of biological samples in case they had a fever of at least 38.5 °C or a known acute infection.

### 2.2. Diagnostic Procedures, Assessments, and Data Collection

Upon recruitment, the participants underwent SPT to a palette of food and inhaled allergens common in Croatia (listed in the Supplementary Material, Table S1).

Standardized SPT controls and inhaled allergen extracts were obtained from Diater Laboratorio de Diagnóstico y Aplicaciones, S.A. (Madrid, Spain). Food allergen extracts were freshly prepared by combining/maceration of fresh or frozen food allergen with 1 mL of saline solution (the prick-to-prick method) [18]. A drop of each solution was applied to the volar forearm surface, and the skin was pricked using a sterile lancet. The negative SPT control was a saline solution, while the positive control was 1 mg/mL histamine hydrochloride. Reactions were measured 15 min post-application. All SPT procedures were done by the same personnel (from the Srebrnjak Children’s Hospital) to avoid inter-operator variability and according to the European Academy of Allergy and Clinical Immunology (EAACI) guidelines [18,19]. Children whose parents/caregivers agreed to it also underwent blood sampling.

Peripheral blood samples were collected by venipuncture into vacutainers with a gel clot activator. Vacutainers with blood samples were centrifuged for 10 min at 3000× *g* for serum separation. Serum samples were stored at −80 °C until further analyses.

### 2.3. Questionnaires and Data Collection

To screen the school-aged and preschool population in Croatia for allergic diseases (personal and family history), lifestyle and dietary habits, several questionnaires were used (socioeconomic questionnaire, ISAAC phase II, questionnaire on customer awareness and perception of micro- and nanoplastics (MNPs), food logs and the food frequency questionnaire—FFQ) [20,21,22,23,24]. A questionnaire on the assessment of the level of exposure to MNPs was developed based on previously reported surveys and the WHO report on the potential implications of exposure to MNPs to human health [22,25] (Supplementary Table S2).

### 2.4. Data Preprocessing and Statistical Analysis

Variables containing string notations were numerically encoded. Allergic sensitization data were expressed as a ratio of the average urticaria wheel diameter of the tested allergen to the urticaria wheel diameter of histamine (positive control) in the SPT (sensitization index). In order to avoid false positives as much as possible, a sensitization index equal to or greater than 0.6 was considered a positive reaction to an allergen [26,27]. Additionally, certain features of allergic sensitization (such as polysensitization) and morbidity (allergic diseases) were converted to binary or ordinal features (yes/no, low/normal/high, etc.).

The Kolmogorov–Smirnov test was used to test the distribution of data for normality. The statistical significance of differences in sensitization rates across demographic groups (sex, age, and region) was evaluated by Chi-Square (χ^2^) tests using cross-tabulations. In cases where assumptions of the Chi-Square test were not met (e.g., expected counts below 5), Fisher’s Exact Test was used. Statistical significance was determined if the obtained *p*-value was <0.05. Since multiple sensitization and food allergy prevalence rate outcomes were considered, the *p*-values of each analysis were interpreted with caution and considered exploratory. Additionally, a false discovery rate (FDR) correction was applied as a sensitivity analysis.

Multivariable logistic regression models were fitted for each sensitization and polysensitization outcome, according to age, sex, and region simultaneously as covariates.

Statistical analysis was performed using the R version R-4.4.3 for Windows [28] and RStudio version 2024.04.2+764 [29], as well as MedCalc^®^ Statistical Software version 23.1.7 [30]. Visualization of prevalence rates was realized using bar charts that were generated using the ggplot2() package in R studio.

## 3. Results

Certain demographic characteristics of the cohorts are shown in Table 1.

In order to determine the sensitization rates to different allergens, sensitization profiles and the food allergy prevalence rates in the Croatian pediatric population, food allergens used in skin prick testing were grouped to align with the categories of reported food allergies. The absolute and relative variability of reported food allergies across regions was assessed, providing insight into the consistency and gender and regional distribution of specific allergen sensitization rates. The participants were further divided into groups by region, sex, and age among preschool- and school-aged children.

**Figure 1:**
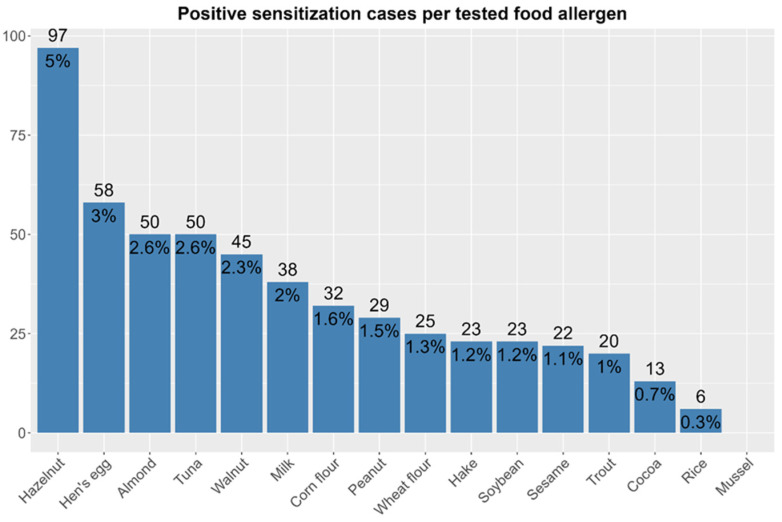
Total sensitization rates to individual food allergens in the study population (n = 1948), according to skin prick test results.

A total of 41% of participants in this study (n = 790) were sensitized to at least one allergen (inhaled or food), while the total sensitization rate to at least one food allergen was 13% (n = 512). Figure 1 shows the total sensitization rates to each food allergen in Croatian children. 

**Figure 2:**
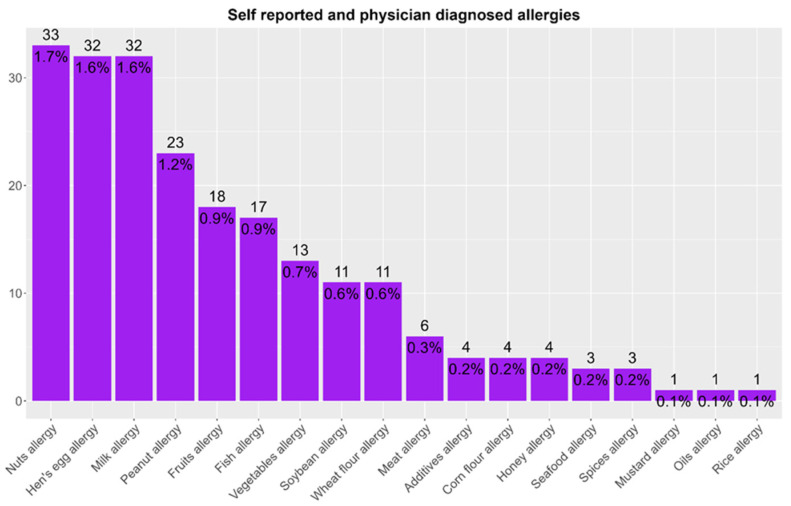
Total food allergy prevalence rates in the study population (N = 1948), according to each food allergen tested. Food allergy to hazelnut, walnut, and almond was grouped into a single category (nut allergy), as was allergy to trout, tuna, and hake (fish allergy).

Figure 2 shows the total food allergy prevalence rates to specific allergens.

Specific allergen sensitization rates significantly differed from corresponding food allergy prevalence rates (Table 2).

In total, 512 (26.27%) participants were polysensitized (to either inhaled or food allergens), while 112 (5.75%) were polysensitized to food allergens.

### 3.1. Age- and Sex-Specific Trends in Sensitization Prevalence Rates

**Figure 3:**
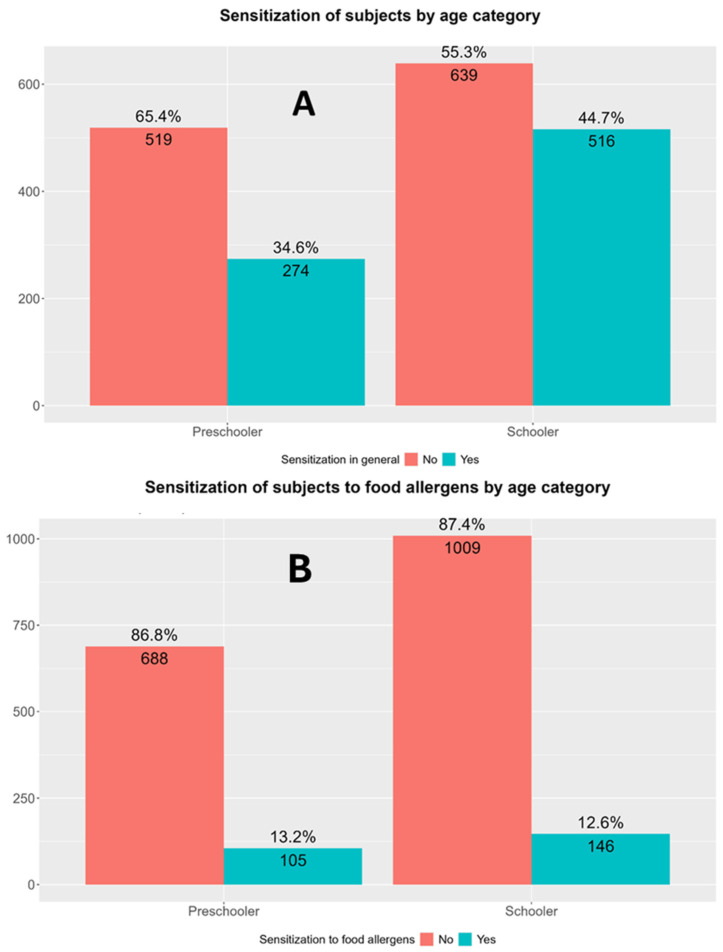
Differences in sensitization rates according to specific age groups. (**A**) Total sensitization rates in preschool- and school-aged children. Pearson’s Chi-Squared Test: χ^2^ = 19.57, *p* < 0.0001, and *p*-value FDR adjusted = 2.238305 × 10^−5^. (**B**) Sensitization rates to food in preschool- and school-aged children. Pearson’s chi-squared test: χ^2^ = 0.10, *p* = 0.7493, and *p*-value FDR adjusted = 0.985. Preschooler—preschool children (1–7 years of age); schooler—school-aged children (6.5–18 years old).

The participants were divided into two groups regarding their age: preschool children (1–7 years) and school-aged children (6.5–18 years). The total sensitization rate was higher in older (school-aged children) compared with preschool participants, while the sensitization rate to food allergens only did not differ between the two age groups (Figure 3).

**Figure 4:**
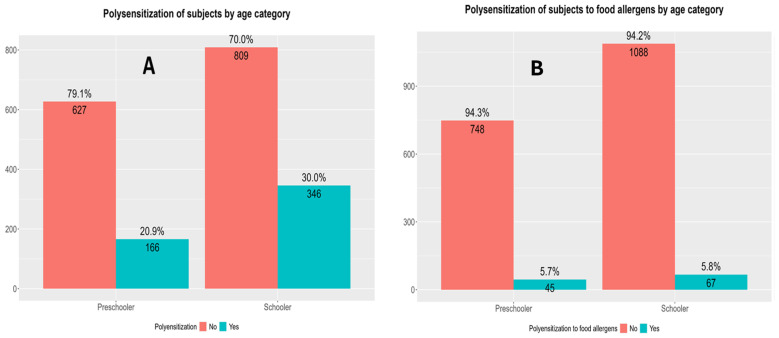
Differences in polysensitization rates (sensitization to two or more allergens) according to specific age groups. (**A**) Differences in total polysensitization rates between preschool- and school-aged children. Pearson’s Chi-Squared Test: χ^2^ = 19.30, *p* < 0.0001, and *p*-value FDR adjusted = 2.238305 × 10^−5^. (**B**) Differences in polysensitization rates to food allergens only between preschool- and school-aged children. Pearson’s chi-squared test: χ^2^ = 0.00034, *p* = 0.985, and *p*-value FDR adjusted = 0.985. Preschooler—preschool children (1–7 years of age); schooler—school-aged children (6.5–18 years old).

The total polysensitization rate was higher in school-aged children compared with preschoolers, while the polysensitization rate to food allergens did not differ between the two age groups (Figure 4).

**Figure 5:**
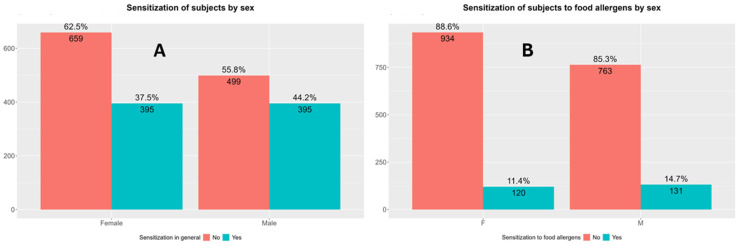
Differences in sensitization profiles according to sex. (**A**) Total sensitization rates in boys and girls. Pearson’s Chi-Squared Test: χ^2^ = 8.75, *p* = 0.003, and *p*-value FDR adjusted = 0.00413. (**B**) Sensitization rates to food allergens in boys and girls. Pearson’s Chi-Squared Test: χ^2^ = 4.32, *p* = 0.038, and *p*-value FDR adjusted = 0.038. F—female children (girls); M—male children (boys).

Both the total sensitization rate and sensitization rate to food allergens were higher in boys compared with girls (Figure 5).

**Figure 6:**
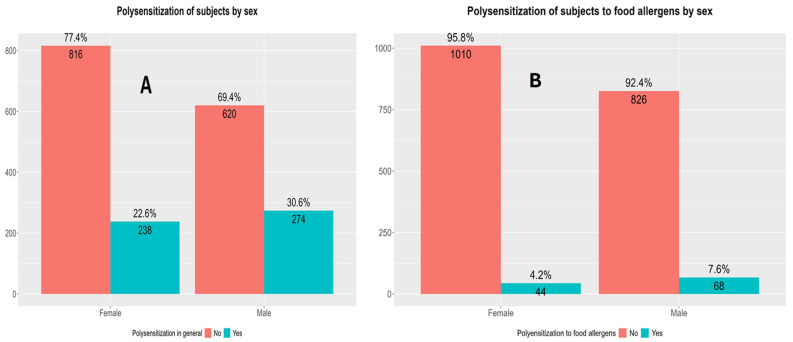
Differences in polysensitization rates (sensitization to two or more allergens) in boys and girls. (**A**) Differences in total polysensitization rates between boys and girls. Pearson’s Chi-Squared Test: χ^2^ = 15.84, *p* < 0.0001, and *p*-value FDR adjusted = 0.00028. (**B**) Differences in polysensitization rates to food allergens only between boys and girls. Pearson’s Chi-Squared Test: χ^2^ = 9.89, *p* = 0.0017, and *p*-value FDR adjusted = 0.0033. F—female (girls); M—male (boys).

Both the total polysensitization rate and polysensitization rate to food allergens were higher in boys compared with girls (Figure 6).

### 3.2. Region-Specific Trends in Sensitization and Food Allergy Prevalence Rates

**Figure 7:**
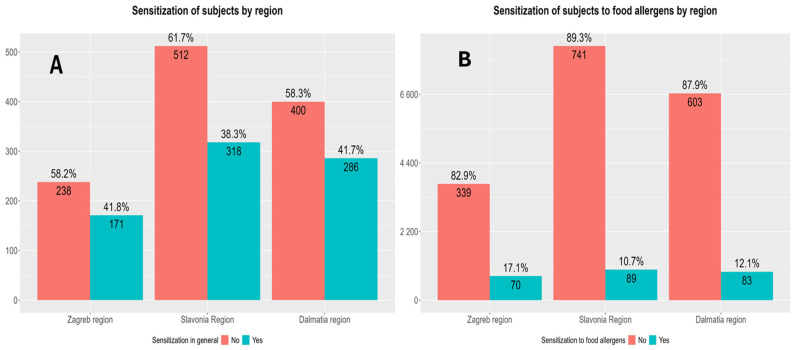
Differences in total sensitization rate and sensitization rate to food allergens according to region of origin. (**A**) Differences in total sensitization rates between different regions of origin: Zagreb (the capital city of Croatia and its surroundings), Dalmatia (Mediterranean region), and the eastern continental region of Slavonia. Pearson’s Chi-Squared Test: χ^2^ = 2.30 and *p* = 0.32. (**B**) Differences in sensitization rates to food allergens only between different regions of origin. Pearson’s Chi-Squared Test: χ^2^ = 10.40 and *p* = 0.0055.

There were no differences in total sensitization rates between different regions of participant origin. However, sensitization rates to food allergens were significantly higher in the Zagreb region (Figure 7).

**Figure 8:**
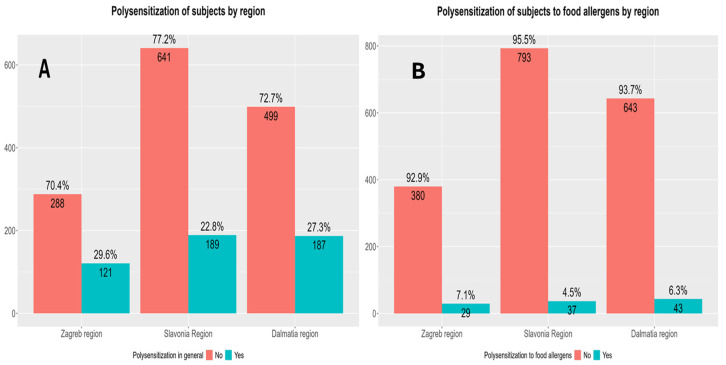
Differences in polysensitization rates (sensitization to two or more allergens) between regions of origin: Zagreb and surroundings, Dalmatia and Slavonia. (**A**) Differences in total polysensitization rates between regions of participant origin. Pearson’s Chi-Squared Test: χ^2^ = 7.80 and *p* = 0.02. (**B**) Differences in polysensitization rates to food allergens only between different regions of participant origin. Pearson’s Chi-Squared Test: χ^2^ = 4.29 and *p* = 0.12.

Total polysensitization rates were higher in the Zagreb region compared with the Slavonia and Dalmatia regions. There were no differences in polysensitization rates to food allergens only between regions of participant origin (Figure 8).

The sensitization rates to specific food allergens and their absolute and relative variability between different regions of participant origin (Zagreb, Slavonia and Dalmatia) are shown in Table 3.

### 3.3. Detected Sensitization Compared with Self-Reported Allergy

FA prevalence rates were assessed as “self-reported” according to the questionnaire in Table S2, while the physician-diagnosed (clinically proven) FA prevalence rate was assessed according to the participants’ medical records. The self-reported FA prevalence rates and detected sensitization to specific foods are shown in Table 4.

**Figure 9:**
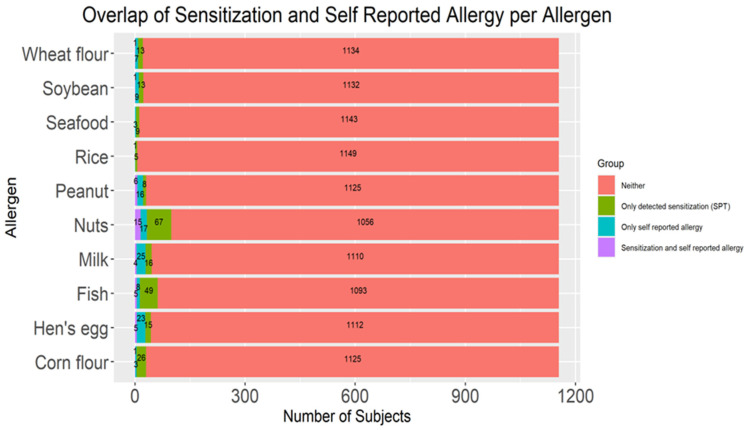
Stacked bar plot with absolute frequencies of detected sensitization and self-reported food allergy for each tested allergen among school-aged children; N = 1155. SPT—skin prick test.

Out of 1948 subjects involved in this study, 1155 schoolchildren had recorded responses to the questionnaire regarding perceived food allergies. Agreement between the sensitization rates and self-reported food allergy prevalence rates for each tested allergen is presented in Table 5 and visualized in Figure 9. Overall agreement between methods was low to moderate, while Cohen’s κ values ranged from −0.004 (seafood) to 0.323 (peanut). The highest overlap between detected sensitization and self-reported allergy was observed for nuts (n = 15), peanut (n = 6) and fish (n = 5), while no overlap was found for rice and seafood. Sensitivity ranged from 0.00 to 0.43 across allergens, whereas specificity was consistently high (≥0.97 for all allergens). Sensitivity and specificity represent conditional proportions of self-reported FA to sensitization detected by SPT and were interpreted as measures of overlap rather than diagnostic performance. Statistically significant discordance between sensitization and self-reported allergy, based on McNemar’s test, was observed for corn flour, fish, and nuts (*p* < 0.001 for all). The odds ratios indicated that participants with self-reported allergy were more likely to show corresponding sensitization for most allergens, with the strongest associations for peanut (OR = 51.56; 95% CI: 13.19–193.75), nuts (OR = 13.83; 95% CI: 6.14–30.89), and fish (OR = 13.85}; 95% CI: 3.43–50.13). Sensitivity was calculated as the proportion of sensitized children who reported allergy, and specificity as the proportion of non-sensitized children who did not report allergy.

**Figure 10:**
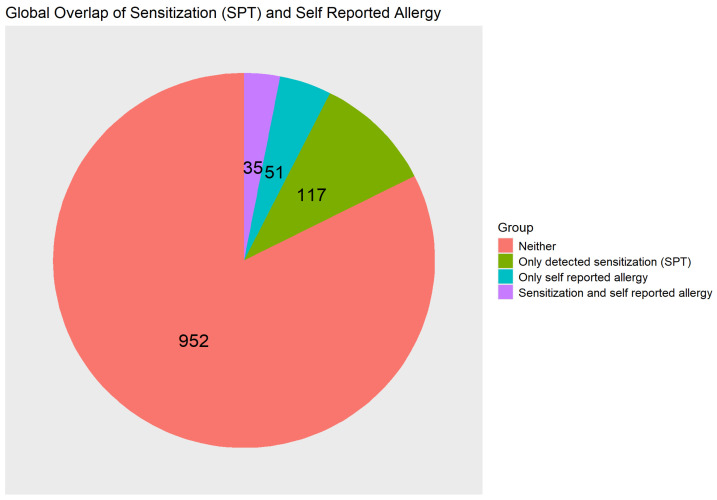
Pie chart showing global alignment between detected sensitization and self-reported food allergy across all tested allergens in school-aged children; N = 1155.

Global analysis across all allergens showed that 35 children (3.0%) exhibited both detected sensitization and self-reported allergy, while 117 (10.1%) were sensitized without reporting symptoms, and 51 (4.4%) reported allergy without corresponding sensitization (Figure 10), with an overall sensitivity of 23% and a specificity of 95%. McNemar’s test indicated that the two measures (sensitization and FA) significantly differed (*p* < 0.001). Sensitized participants were more likely to report a specific food allergy (sensitized to the corresponding allergen) compared with non-sensitized individuals (OR = 5.571; 95% CI = 3.368–9.146); however, the overall agreement was low (κ = 0.22).

### 3.4. Sensitization Rates Compared with Physician-Diagnosed Allergy

Out of 793 preschool children, data on physician-diagnosed food allergies were available and compared with sensitization detected via SPT (Table 6). Sensitization was most frequently detected for nuts (5.29%) and hen’s egg (4.79%). Physician-diagnosed allergies were reported most frequently for fish (0.50%), hen’s egg (0.50%), milk (0.38%), and wheat/gluten (0.38%), while all other diagnosed allergies were reported in ≤ 0.13% of preschool participants. There were no differences in the distribution of detected sensitization rates and physician-diagnosed food allergies across all tested allergens.

**Figure 11:**
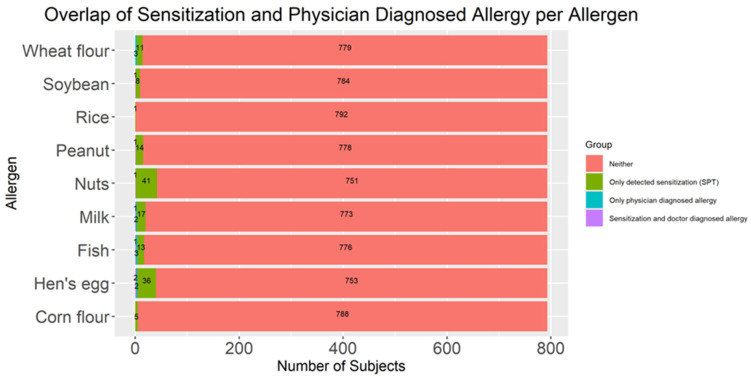
Stacked bar plot with absolute frequencies of detected sensitization and physician-diagnosed food allergy for each tested allergen among preschool children; N = 793. SPT—skin prick test.

The agreement between detected sensitization and physician-diagnosed allergy for each allergen is presented in Table 7 and visualized with a stacked bar plot (Figure 11). Sensitization rates for nuts (n = 56), hen’s egg (n = 38), and milk (n = 18) were substantially higher than physician-diagnosed allergy to these foods (n = 1, 4, and 3, respectively). The overall agreement was low to moderate, with Cohen’s κ-values ranging from −0.006 (wheat) to 0.198 (soybean). The highest overlap between detected sensitization and physician-diagnosed allergy was observed for hen’s egg (N = 2), while one positive case was detected for milk, soybean, peanut, fish and nuts. For other tested food allergens, there was no overlap between detected sensitization and physician-diagnosed allergy. Sensitivity (calculated as the proportion of sensitized children who had a physician-diagnosed allergy) ranged from 0 to 11.1% across allergens, whereas specificity (the proportion of non-sensitized children without diagnosed allergy) was consistently high (≥99.6% for most allergens). Statistically significant discordance, based on McNemar’s test, was observed for hen’s egg (*p* < 0.001), nuts (*p* < 0.001), peanut (*p* < 0.001), milk (*p* = 0.001), soybean (*p* = 0.0133), and fish (*p* = 0.024). Children with detected sensitization to a specific allergen were more likely to have a corresponding physician-diagnosed allergy for most allergens, with the strongest associations for milk (OR = 22.34; 95% CI: 0.36–447.28), hen’s egg (OR = 20.65; 95% CI: 1.46–291.34), and fish (OR = 19.56; 95% CI: 0.35–262.94). For several allergens (soybean, peanut, nuts, corn, and rice), no children had a physician-diagnosed allergy without corresponding sensitization, resulting in odds ratios that were infinite (OR = ∞).

**Figure 12:**
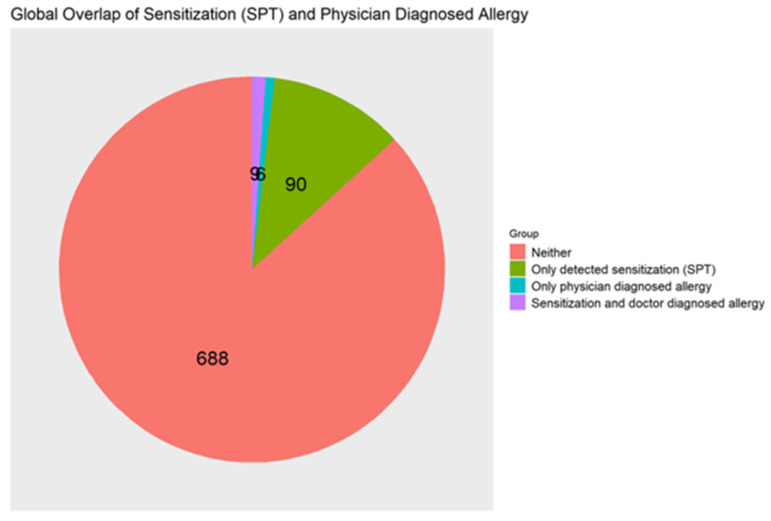
Pie chart showing global alignment between detected sensitization and physician-diagnosed food allergy across all tested allergens in the preschool population; N = 793.

Figure 12 shows the global overlap of sensitization and physician-diagnosed food allergy prevalence rates, with 1.1% of participants being both sensitized and with a physician-diagnosed allergy to a specific food, and 11.4% were only sensitized but did not have a corresponding food allergy diagnosis, and 0.76% had a physician-diagnosed food allergy without being sensitized to the corresponding allergen. The overall sensitivity of physician-diagnosed allergy compared with detected sensitization was 9%, and the specificity was 99%. McNemar’s test indicated a statistically significant difference between the two measures (*p* = 2.43 × 10^−17^). Again, the specificity and sensitivity here represent conditional proportions of physician-diagnosed FA to sensitization and indicate measures of overlap. Children with detected sensitization were more likely to have a corresponding physician-diagnosed FA compared with non-sensitized children (OR = 11.40; 95% CI: 3.53–39.89); however, the overall level of agreement was low (κ = 0.13).

### 3.5. Multivariable Analyses of Sensitization Rates

Multivariable logistic regression analyses of sensitization rates, accounting for region simultaneously, were conducted for each outcome. A total of 23 participants (1.2%) were excluded due to missing data on the region.

For total sensitization rates, school-aged children had significantly higher odds compared with preschool-aged children. Female sex was associated with a lower risk of being sensitized to one or more allergens compared with male sex. There were no significant differences in the risk for total (global) sensitization between regions of participant origin (Table 8).

School-aged children had markedly higher odds for being polysensitized to at least one allergen compared with preschoolers, and girls had lower odds compared with boys. Regional differences were partially observed: the odds for polysensitization in general were significantly lower in the Slavonia region compared with Zagreb, while the Dalmatia and Zagreb regions did not differ from each other (Table 9).

For sensitization to food allergens, no significant differences were observed according to the age category. Female sex was associated with lower odds compared with male sex. Additionally, participants in both the Slavonia and Dalmatia regions had lower odds of being sensitized to one or more food allergens compared with the Zagreb region (Table 10).

For polysensitization rates to food allergens, there were no differences in the odds ratios between the two age categories. Female sex remained strongly associated with lower odds for polysensitization to food. As for regional differences, the odds were lower in the Slavonia region compared with Zagreb, while Dalmatia did not differ significantly (Table 11).

## 4. Discussion

The results of this study provide a comprehensive overview of allergen sensitization patterns among children in three regions of Croatia, offering insights into age-, gender-, and region-specific trends. Our findings demonstrate that sensitization to one or more allergens was present in 41% of participants. This rate indicates a substantial burden of allergic sensitization in childhood, and such trends have also been previously published in epidemiological studies [31,32,33]. Sensitization rates to food allergens were lower than the sensitization rates to inhaled allergens, but still relatively high (13%), highlighting the growing relevance of food allergy surveillance in children [34,35,36,37,38].

Hazelnut was the most common sensitizer, affecting 5% of the children. Other tree nuts, such as almond (2.6%) and walnut (2.3%), also ranked high, suggesting that tree nuts represent a major allergen source. This aligns with patterns observed in Europe, where tree nuts and hen’s egg are one of the most common triggers of allergy in early life [34,35]. Sensitization to hen’s egg (3%) and milk (2%) was also relatively common, consistent with their status as leading childhood allergens globally [36,37], while sensitization to tuna (2.6%) also appeared as common, indicating it is an important allergenic source in Croatian children.

The sensitization rates for hen’s egg and milk were the second and third highest documented in this study (3% and 2%, respectively), while the self-reported allergy to hen’s egg and milk (both 1.6%) along with physician-diagnosed allergy to hen’s egg and milk prevalence rates were also in the top three reported (both 0.2%). Interestingly, the sensitization rate to peanut was lower compared with other food allergens such as cow’s milk, hen’s egg, nuts, and fish (1.5%). However, self-reported and physician-diagnosed peanut allergy prevalence rates were among the highest (1.9% and 0.13%, respectively). This discrepancy could be the result of heightened awareness among healthcare providers regarding the risks associated with peanut allergy, which is often linked to severe reactions and is, therefore, more likely to be diagnosed and reported [38].

The sensitization rates to fish species such as tuna and hake (commonly consumed in Croatia) were relatively high (2.6% and 1.2%, respectively). Since parvalbumin, the major allergen in fish is known to elicit cross-reactivity among different fish species as it is highly conserved [39], both sensitization rates and allergy prevalence rates to tuna, trout, and hake were combined into a single category, fish, with combined 93 positive sensitization cases (4.77%), while fish allergy was diagnosed in four (0.2%) participants. This indicates that the highest physician-diagnosed allergy prevalence rate was that for fish, alongside hen’s egg allergy. However, there was a discrepancy in the sensitization rate to fish and the self-reported allergy prevalence rate to it, and in the case of self-reported allergy (13 participants (0.7%)), indicating that allergy to fish is less recognized (reported) than allergy to other food sources, such as milk, nuts, hen’s egg, peanut and fruits.

A significant discrepancy between detected sensitization rates and self-reported allergy prevalence rates was observed with nuts: 192 children were sensitized, but only 33 were reported to have a nut allergy (*p* = 0.0004998), underlining the well-known fact that sensitization does not always translate into clinically manifested allergy [36]. Several factors may contribute to this mismatch, including a lack of clinical evaluation following a positive test, variable symptom perception among patients and their caregivers, or limitations in diagnostic accessibility [40].

Polysensitization was common in the Croatian pediatric population, with 26% of the participants sensitized to two or more allergens, but only 5.7% were polysensitized to food allergens. Nonetheless, the subset of polysensitized children to food allergens may represent a group at higher risk for developing more complex or persistent allergic diseases [41].

While the overall sensitization rates were comparable across the three investigated regions, with Zagreb and Dalmatia showing nearly identical frequencies (41.7% and 41.8%, respectively) and Slavonia showing a slightly lower one (38.3%), when focusing on food allergens, regional disparities were more evident. Sensitization rates to food allergens were higher in the Zagreb region (17.1%) compared with Dalmatia (12.1%) and Slavonia (10.7%). This finding may suggest that dietary habits, urban lifestyle, or other region-specific environmental exposures are more different and more pronounced in the Zagreb area, increasing the risk for allergic sensitization [42]. Additionally, these differences might have been influenced by the protective environmental factors associated with rural residence (such as farming environment), as the majority of participants residing in rural areas originated from the Slavonia and Dalmatia regions [43].

Hazelnut was the most prevalent allergen, with consistent sensitization rates across all three regions (CV = 0.12). Other allergens, such as hen’s egg, tuna, almond, corn flour, soybean, and trout, displayed high variability in sensitization rates between regions. These findings may indicate regional heterogeneity in exposure or sensitization patterns for specific foods; however, the overall distribution of sensitization rates to individual food allergens was not significantly different between regions (*p* = 0.101), implying that while sensitization to individual allergens varies by region, the general pattern is not predominantly region-specific.

In contrast with sensitization rates, food allergy prevalence rates were significantly different between regions (Figure 7). The most common allergies were those to nuts, hen’s egg, and milk, and their prevalence rates varied to a certain extent across regions. Notably, nuts and hen’s egg allergies were more common in the Zagreb region, while milk allergy was more common in Slavonia and Dalmatia. High relative variability in the reporting of less common allergies, such as those to additives, corn flour, spices, mustard, oils and rice, emphasizes the regional differences in either true prevalence or diagnostic/reporting practices. It is possible that some of this variation is not influenced only by regional dietary habits but also by access to healthcare and awareness issues in underpinning the culprit food or other factors associated with the “hygiene hypothesis” [43,44].

General polysensitization rates also showed statistically significant variation across tested regions, with the highest rate documented in the Zagreb region (29.6%) and the lowest in Slavonia (22.8%). This trend may point to a greater cumulative sensitization burden in urban or more industrialized areas. Contrary to general polysensitization, polysensitization rates to food allergens did not differ significantly across regions. Nevertheless, the consistently higher polysensitization rates in Zagreb and Dalmatia suggest a potential influence of local dietary habits and exposure patterns [45], though further investigation would be required to confirm these associations.

The analysis of sensitization patterns between sexes in the studied pediatric population revealed significant differences in overall sensitization rates, as well as in sensitization rates to food allergens and distinct patterns of polysensitization. These findings align with prior research suggesting a sex-specific vulnerability to allergic sensitization for both inhalant and food allergens, particularly in early life stages [46,47,48].

The results of this study show that the total sensitization rate was higher in boys compared with girls (44.2% vs. 37.5%; *p* = 0.001), consistent with previous findings reporting a male predominance in sensitization rates to both food and inhaled allergens, as well as in allergic diseases prevalence rates during childhood, particularly in asthma, atopic dermatitis, and allergic rhinitis [46,47,48]. One potential explanation for this disparity lies in sex-based immunological and hormonal differences, particularly in the period before puberty, when males typically display a more reactive Th2-skewed immune profile, predisposing them to allergic inflammatory immune responses [49,50]. Boys also exhibited higher sensitization rates to food allergens compared with girls (14.7% vs. 11.4%; *p* = 0.037). This observed sex difference may further underscore the heightened immunological responsiveness among boys or could reflect differential environmental exposures, dietary habits, or microbial colonization in early life. However, the differences in sensitization rates to food allergens were less pronounced than those with total sensitization rates, which may be the natural course of food allergies, many of which (e.g., to milk or hen’s egg) resolve in early childhood [51,52,53]. Polysensitization was also more prevalent in boys (30.7%) compared with girls (22.6%; *p* < 0.001). This finding is particularly relevant, as polysensitization has been associated with increased disease severity, persistence, and a broader atopic burden [53]. This gap between sexes persisted when looking into polysensitization to food allergens only, with 7.6% of boys and 4.2% of girls being polysensitized to food (*p* = 0.002), again likely reflecting the proposed heightened allergic immunological response among male children [50,51,52].

The analysis of age-specific trends in allergic sensitization within the study population revealed significant differences in total sensitization and polysensitization rates between preschool and school-aged children. Children in the school-aged group exhibited a higher total sensitization rate (44.8%) compared with preschool children (33.4%; *p* < 0.001). This trend is in line with existing evidence indicating a cumulative effect of environmental exposures over time, such as increased contact with aeroallergens, changes in lifestyle, and maturation and modulation of the immune system during childhood. As children grow older, their immune systems mature and may become increasingly responsive to a broader range of allergens, particularly inhalant allergens, which typically drive the rise in sensitization rates in school-aged populations [54].

In contrast, sensitization to nutritive allergens was observed at nearly identical rates in both preschool (13.3%) and school-aged children (12.4%; *p* = 0.625). This finding may reflect the natural history of sensitization to food allergens, which tends to occur early in life, often within the first years due to dietary introduction of common allergens [55]. It is also possible that some early sensitizations may resolve with age, especially in the case of milk or hen’s egg allergy, leading to a relative plateau in prevalence across age groups [56]. Total polysensitization follows the same trend as for the sensitization rates between the two age groups, with 29.8% of school-aged children being polysensitized compared with 20.5% of preschoolers (*p* < 0.001). This suggests that polysensitization, like gross sensitization, may accumulate over time as children are exposed to a wider array of allergens, especially in predisposed individuals [57]. However, when focusing solely on polysensitization rates to food allergens, these did not differ in the two age groups (5.9% in preschoolers and 5.6% in school-aged children; *p* = 0.919). This further reinforces the notion that sensitization to food allergens, particularly to multiple different foods, does not progress significantly with age, potentially due to natural tolerance development during immune system maturation or limited new exposure to novel dietary proteins during these age stages [58].

Comparison of sensitization rates and self-reported allergy for each allergen revealed notable differences across several foods. Although 13% of schoolchildren were sensitized to at least one food allergen, only 8.3% reported a food allergy, highlighting the frequent mismatch between detected sensitization and clinical manifestation of food allergy. This is consistent with previously published findings showing that positive SPT results do not always correspond to clinically relevant allergy [40]. The results of this study show that nuts and fish were the most common sensitizers (11.8% and 6.6%, respectively), yet they were substantially less frequently self-reported as allergenic (2.8% and 1.1%). Conversely, milk, hen’s egg, and peanut allergies were more often self-reported than detected via SPT, suggesting either a heightened awareness and reporting bias for these allergens or even the development of tolerance in some previously sensitized children.

Despite these individual discrepancies, there was a significant overall discordance between detected sensitization rates and self-reported allergy (*p* < 0.001), with only 3% of participants showing concordance between the two measures. The overall agreement was low (κ = 0.22), while specificity was high (95%), indicating that most non-sensitized children did not report food allergies. Additionally, children with sensitization were over five times more likely to report food allergy compared with non-sensitized peers. These findings align with evidence suggesting that self-reported allergy tends to overestimate true food allergy prevalence rates, reflecting patient/caregiver perception and bias, mild or past symptoms, and the heterogeneity in healthcare availability and confirmatory diagnostic procedures [9,58]. Moreover, certain conditions, such as food intolerance, can be mistaken and falsely reported as food allergy, leading to the overestimation of food allergy prevalence rates. A certain proportion of participants who have non-IgE-mediated food allergy may also contribute to the discrepancies between sensitization and self-reported food allergy prevalence rates [58].

A similar pattern was observed when sensitization data were compared with physician-diagnosed food allergy among preschool children. Although sensitization was detected in 12,5% of subjects, only 1.9% had a documented diagnosis of food allergy, underscoring an even wider gap between positive SPT results and clinically relevant food allergy in this younger cohort. Sensitization rates to hen’s egg (4.8%), nuts (7.1%), and milk (2.3%) were notably higher than the corresponding physician-diagnosed food allergy prevalence rates (0.5%, 0.1%, and 0.4%, respectively). A significant overall discordance was also found between the different sensitization rates and physician-diagnosed food allergy prevalence rates (*p* < 0.001), further supporting the notion that sensitization alone is not a sufficient indicator of clinical allergy [58]. The agreement between SPT results and confirmed diagnosis of food allergy was even lower than that with self-reported allergy (κ = 0.13), with sensitivity being 9% and specificity of 99%, which indicates that nearly all non-sensitized children were correctly classified as non-allergic, but at the same time, most sensitized children lacked a formal diagnosis. Only 1.1% of preschoolers were both sensitized and had a physician-diagnosed food allergy. The global odds ratio showed that sensitized children were over eleven times more likely to have a clinically relevant food allergy compared with non-sensitized peers. For several allergens—including soybean, peanut, nuts, corn, and rice—no physician-diagnosed allergies were reported in non-sensitized children, resulting in infinite odds ratios, suggesting perfect but rare concordance within this small sample.

The sensitization rates to food allergens were similar in both age groups (13% in school-aged and 12.5% in preschool children). Overall, our multivariable analyses indicate that age and sex are consistently associated with total sensitization and polysensitization rates, with school-aged children and boys having higher odds. In contrast, regional differences were more evident for sensitization to food allergens and polysensitization outcomes, suggesting that environmental or dietary factors may play a role in these patterns. Importantly, the persistence of age and sex effects after adjustment for region suggests that these associations are not simply due to regional differences and are likely to reflect intrinsic biological or developmental factors. These findings align with previous studies showing that sensitization patterns vary by age and sex, while environmental exposures contribute more specifically to allergen-type-specific outcomes [43].

The levels of agreement between the sensitization FA prevalence rates in self-reported allergy and physician-diagnosed allergy were low and differed between each other (κ = 0.22 vs. 0.13, respectively). One study involving a pediatric population in Croatia reported a parental/caregiver-reported FA prevalence rate of 13.5%; however, the study population involved infants and young preschool children [12]. FA is less common in older children, and the results of our study (showing a self-reported FA prevalence rate of 8.3%) are in concordance with previous findings [56]. Meanwhile, the study involving a Croatian infant and early preschool population reported a clinically proven FA prevalence rate of 5.4% [12], while our study reports a much lower prevalence rate (1.9%). This may be due to certain differences in the populations between the studies. Our study involved children up to the age of 7 years, and infants were not involved, while Voskresensky Baricic et al. [12] involved younger children (6–48 months of age). Additionally, since the participants in our study were recruited through preschool institutions in the three Croatian regions, these discrepancies in FA prevalence rates may also be due to differences in preschool availability and practices countrywide, as smaller places and rural areas may have limited access to preschool education or in general, preschool institutions enroll children with clinically proved FA at a different rate in different town and regions.

The limitations of this study mainly lie in the differences in reporting food allergy prevalence rates between the two age groups, with self-reported allergy in school-aged children and physician-diagnosed allergy in the preschool population. Although both concepts are valid and have certain advantages, they may also lead to over- or underestimation of food allergy prevalence rates due to self-reporting relying on the subjective perception of the patient/caregiver, which may mistake similar symptoms of other conditions (such as food intolerances) with food allergy and, on the other hand, physician-confirmed diagnosis often requiring a substantial amount of time to be established and healthcare (including allergy testing, diagnostic procedures and allergy specialists) not being universally available, especially in rural areas. Moreover, the discrepancy between self-reported and medically confirmed allergies underscores the importance of educational and diagnostic strategies to prevent overreporting and underdiagnosis in different age groups. These discrepancies in sensitization rates and food allergy prevalence rates (self-reported vs. physician-diagnosed) may also be due to immune system maturation and modulation during early childhood, with the burden of food allergy being the highest in infancy and preschool age [59]. Moreover, the low physician-diagnosed FA prevalence rate we report may also stem from the study concept that involved preschool institutions, which have different practices countrywide. For example, not all preschool institutions have the facilities, know-how, and logistics to enroll children with FA [17], which is why our study may have underestimated the clinically confirmed FA prevalence rates. Additionally, the study results may have been influenced by the differences in sample sizes in different subgroups of participants (preschoolers vs. school-aged children; participants from the Zagreb and Mediterranean regions vs. participants in the Slavonia region). However, the sample sizes in each subgroup were substantial, and any bias in the recruitment process was mitigated by the cross-sectional nature of the study design and by recruiting all participants who did not meet the exclusion criteria. Moreover, using a sensitization index as a criterion for determining sensitization is less commonly used in clinical studies on FA, compared with an urticaria wheal diameter greater than 3 mm. However, as sensitization is often known not be clinically relevant, using a stricter criterion (such as a sensitization index of 0.6 according to the histamine urticaria wheal size) might improve specificity in SPT [60]. Lastly, the agreement analysis was designed to examine the clinical relevance of sensitization to certain food allergens detected by SPT compared with FA rather than to validate SPT as a diagnostic test in establishing a diagnosis of FA. In the absence of a definitive gold standard for food allergy, such as the oral food challenge, neither sensitization nor a previously established physician diagnosis of FA can be considered a true reference standard. Consequently, sensitivity and specificity in our study were used descriptively to quantify conditional overlap between sensitization and clinically proven or self-reported allergy. Low Cohen’s κ-values should be interpreted cautiously, considering the context of very low prevalence of physician-diagnosed FA and asymmetric marginal distributions, conditions under which κ is known to underestimate agreement despite high specificity. However, despite these limitations, κ statistics, McNemar’s test, and conditional sensitivity/specificity were retained to provide a comprehensive description of agreement and discordance patterns between sensitization and reported FA.

The findings of this study emphasize the complex relationship between allergic sensitization, perceived symptoms, and clinically relevant disease manifestations in pediatric food allergy. The relatively low agreement observed between sensitization ratings and food allergy prevalence rates supports the need for confirmatory diagnostic testing, such as oral food challenges, to accurately distinguish between sensitization and true and clinically relevant allergy. These findings also highlight the complex interplay of environmental, biological (intrinsic) and regional factors in pediatric allergic sensitization and emphasize the requirement for targeted public health strategies, improved diagnostic pathways, and continued surveillance to inform allergy prevention and management efforts in children. Understanding regional and dietary influences on sensitization patterns, as well as exploring genetic and environmental risk factors, may also contribute to more personalized allergy prevention and management strategies. Finally, further research involving both self-reported and physician-diagnosed food allergy prevalence rates, large cohort sizes, and preferably multi-center and longitudinal studies is required to confirm the results of this study.

## 5. Conclusions

Our study provides a comprehensive overview of allergen sensitization and food allergy patterns in Croatian children, demonstrating a high overall burden of allergic sensitization (with over 40% of children sensitized to one or more allergens), and notable differences according to sex, age, and region. Sensitization to inhaled allergens was more frequent than to food; however, food allergen sensitization affected a substantial proportion of children (13%), with tree nuts—particularly hazelnut—along with hen’s egg, milk, and fish identified as the most relevant food allergens.

A major finding is the significant discrepancy between allergen sensitization and both self-reported and physician-diagnosed food allergy, indicating that sensitization does not necessarily translate into clinically relevant disease. Although sensitized children had markedly higher odds of reporting or having a diagnosed food allergy, the level of agreement between SPT results and clinical outcomes was low. These results highlight the limitations of relying solely on sensitization data and underscore the importance of confirmatory diagnostic procedures in pediatric allergy assessment.

Polysensitization was also substantial (with 26% of children being sensitized to at least two or more allergens) and more common in boys, school-aged children, and children from more urbanized regions, suggesting a cumulative sensitization burden influenced by environmental and immunological factors. In contrast, sensitization to food allergens and food-related polysensitization appeared early in life and remained relatively stable across age groups, consistent with the natural history of childhood FA and the development of immune tolerance.

In conclusion, this study emphasizes the complexity of pediatric FA and the need for standardized diagnostic approaches to accurately distinguish between sensitization and true food allergy. The observed demographic and regional differences, together with the low concordance between sensitization and clinically confirmed allergy, support the need for improved awareness and further large-scale, multicenter longitudinal studies involving both laboratory in vitro and in vivo diagnostic tests to better define the prevalence and clinical relevance of food allergy in children.

## Figures and Tables

**Table 1:** Basic demographic features (age and sex) of the study participants. Two participants (0.1%) had missing data for sex (1 preschool and 1 school-aged child). SD—standard deviation; y—years.

	Male—N (%)	Female—N (%)	Age—Mean in y (SD)
Total population	893 (45.82)	1054 (54,08)	8.09 (4.73)
Preschool children	422 (53.22)	370 (46.66)	4.33 (1.49)
School-aged children	471 (40.74)	684 (59.17)	11.95 (3.31)

**Table 2:** Comparison of specific allergen sensitization rates with self-reported food allergy prevalence rates and physician-diagnosed food allergy prevalence rates corresponding to each allergen. Allergy to clams, shellfish, crab, prawns, etc. (due to low frequency), was grouped into a single category: seafood allergy. Fisher’s Exact Test and Monte Carlo simulation (1,000,000 replicates): *p* < 0.001.

Food Allergen	Sensitization Rate, N (%; 95% CI)	FA Prevalence Rate (Self-Reported), N (%; 95% CI)	FA Prevalence Rate (Physician-Diagnosed), N (%; 95% CI)
Cow’s milk	38 (1.95%, 95% CI: 1.42–2.67)	29 (1.49%, 95% CI 1.75–3.58)	3 (0.15%, 95% CI 0.13–1.11)
Hen’s egg	58 (2.98%, 95% CI: 2.31–3.83)	28 (1.44%, 95% CI 1.68–3.48)	4 (0.21%, 95% CI 0.2–1.29)
Wheat flour	25 (1.28%, 95% CI 0.87–1.89)	8 (0.41%, 95% CI 0.35–1.36)	3 (0.15%, 95% CI 0.13–1.11)
Soybean	23 (1.18%, 95% CI 0.79–1.77)	10 (0.51%, 95% CI 0.47–1.59)	1 (0.05%, 95% CI 0.02–0.71)
Peanut	29 (1.49%, 95% CI 1.04–2.13)	22 (1.13%, 95% CI 1.26–2.87)	1 (0.05%, 95% CI 0.02–0.71)
Corn flour	32 (1.64%, 95% CI 1.17–2.31)	4 (0.21%, 95% CI 0.13–0.89)	0 (0%, 95% CI 0–0.48)
Rice	6 (0.31%, 95% CI 0.14–0.67)	1 (0.05%, 95% CI 0.02–0.49)	0 (0%, 95% CI 0–0.48)
Nuts	192 (9.9%, 95% CI 8.61–11.26)	32 (1.64%, 95% CI 1.97–3.88)	1 (0.05%, 95% CI 0.02–0.71)
Fish	93 (4.8%, 95% CI 3.91–5.81)	13 (0.67%, 95% CI 0.66–1.92)	4 (0.21%, 95% CI 0.2–1.29)
Seafood	0 (0%, 95% CI 0–0.2)	3 (0.15%, 95% CI 0.09–0.76)	0 (0%, 95% CI 0–0.48)

**Table 3:** Distribution of sensitization rates to specific food allergens and their variability (SD and CV) between different regions of origin; N = 1925. CV_abs_—absolute coefficient of variation, CV_rel_—relative coefficient of variation, SD_abs_—absolute standard deviation, and SD_rel_—relative standard deviation.

Food Allergen	Zagreb—N (%)	Slavonia—N (%)	Dalmatia—N (%)	SD_abs_ (SD_rel_)	CV_abs_ (CV_rel_)
Hazelnut	34 (8.31)	32 (3.86)	27 (3.94)	3.61 (2.54)	0.12 (0.47)
Hens egg	17 (4.16)	15 (1.81)	25 (3.64)	5.29 (1.25)	0.28 (0.39)
Almond	19 (4.65)	20 (2.41)	8 (1.17)	6.66 (1.72)	0.42(0.63)
Tuna	12 (2.93)	25 (3.01)	11 (1.60)	7.81 (0.78)	0.49 (0.31)
Walnut	11 (2.69)	13 (1.57)	17 (2.48)	3.06 (0.59)	0.22 (0.26)
Cow’s milk	11 (2.69)	14 (1.69)	12 (1.75)	1.53 (0.58)	0.12 (0.28)
Corn flour	7 (1.71)	17 (2.05)	7 (1.02)	5.77 (0.51)	0.56 (0.33)
Peanut	11 (2.69)	5 (0.60)	10 (1.46)	3.21 (1.05)	0.37 (0.66)
Wheat flour	9 (2.20)	9 (1.08)	6 (0.87)	1.73 (0.7)	0.22 (0.5)
Hake	5 (1.22)	8 (0.96)	10 (1.46)	2.52 (0.25)	0.33 (0.2)
Soybean	11 (2.69)	4 (0.48)	7 (1.02)	3.51 (1.15)	0.48 (0.82)
Sesame	8 (1.96)	8 (0.96)	5 (0.73)	1.73 (0.68)	0.25 (0.55)
Trout	3 (0.73)	11 (1.33)	6 (0.87)	4.04 (0.31)	0.61 (0.32)
Cocoa	4 (0.98)	6 (0.72)	3 (0.44)	1.53 (0.3)	0.35 (0.43)
Rice	3 (0.73)	2 (0.24)	1 (0.15)	1 (0.32)	0.5 (0.96)
Mussels	0 (0)	0 (0)	0 (0)	0 (0)	0 (0)

**Table 4:** Frequencies (absolute and relative) of detected sensitization (SPT) and self-reported allergies to food allergens. N = 1155; Fishers’ Exact Test: *p* value = 1 × 10^−6^.

Allergy	Detected Sensitization—N (%; 95% CI)	Self Reported Allergy—N (%; 95% CI)
Cow’s milk	20 (1.73%, 95% CI 1.12–2.66)	29 (2.51%, 95% CI 1.75–3.58)
Hen’s egg	20 (1.73%, 95% CI 1.12–2.66)	28 (2.42%, 95% CI 1.68–3.48)
Wheat flour	14 (1.21%, 95% CI 0.72–2.02)	8 (0.69%, 95% CI 0.35–1.36)
Soybean	14 (1.21%, 95% CI 0.72–2.02)	10 (0.87%, 95% CI 0.47–1.59)
Peanut	14 (1.21%, 95% CI 0.72–2.02)	22 (1.9%, 95% CI 1.26–2.87)
Corn flour	27 (2.34%, 95% CI 1.61–3.38)	4 (0.35%, 95% CI 0.13–0.89)
Rice	5 (0.43%, 95% CI 0.19–1.01)	1 (0.09%, 95% CI 0.02–0.49)
Nuts	136 (11.77%, 95% CI 10.04–13.76)	32 (2.77%, 95% CI 1.97–3.88)
Fish	76 (6.58%, 95% CI 5.29–8.16)	13 (1.13%, 95% CI 0.66–1.92)
Seafood	9 (0.78%, 95% CI 0.41–1.47)	3 (0.26%, 95% CI 0.09–0.76)

**Table 5:** Allergen-specific metrics between detected sensitization and self-reported food allergy for individual allergens among schoolchildren; N = 1155.

Allergen	McNemar’s *p*-Value	Cohen’s Kappa (κ)	Odds Ratio	Odds Ratio, Lower	Odds Ratio, Upper	Sensitivity	Specificity
Hen’s egg	0.256	0.192	15.977	4.188	51.479	0.250	0.980
Cow milk	0.212	0.146	11.029	2.505	37.667	0.200	0.978
Wheat flour	0.264	0.083	12.360	0.257	108.671	0.071	0.994
Soybean	0.522	0.074	9.613	0.205	79.028	0.071	0.992
Peanut	0.153	0.323	51.560	13.192	193.754	0.429	0.986
Corn flour	0.000	0.059	14.293	0.265	185.473	0.037	0.997
Fish	0.000	0.134	13.845	3.434	50.129	0.093	0.993
Seafood	0.149	−0.004	0.000	0.000	333.410	0.000	0.997
Nuts	0.000	0.233	13.826	6.142	30.892	0.183	0.984
Rice	0.221	−0.001	0.000	0.000	7867.451	0.000	0.999

**Table 6:** Frequencies (relative and absolute count) of detected sensitization (SPT) and physician-diagnosed allergy to food; N = 793. *p* = 0.112.

Allergy	Detected Sensitization—N (%; 95% CI)	Physician-Diagnosed Allergy—N (%; 95% CI)
Cow’s milk	18 (2.27%, 95% CI 1.44–3.56)	3 (0.38%, 95% CI 0.13–1.11)
Hen’s egg	38 (4.79%, 95% CI 3.51–6.51)	4 (0.5%, 95% CI 0.2–1.29)
Wheat flour	11 (1.39%, 95% CI 0.78–2.47)	3 (0.38%, 95% CI 0.13–1.11)
Soybean	9 (1.13%, 95% CI 0.6–2.14)	1 (0.13%, 95% CI 0.02–0.71)
Peanut	15 (1.89%, 95% CI 1.15–3.1)	1 (0.13%, 95% CI 0.02–0.71)
Corn flour	5 (0.63%, 95% CI 0.27–1.47)	0 (0%, 95% CI 0–0.48)
Rice	1 (0.13%, 95% CI 0.02–0.71)	0 (0%, 95% CI 0–0.48)
Nuts	56 (7.06%, 95% CI 5.48–9.06)	1 (0.13%, 95% CI 0.02–0.71)
Fish	17 (2.14%, 95% CI 1.34–3.41)	4 (0.5%, 95% CI 0.2–1.29)
Seafood	0 (0%, 95% CI 0–0.48)	0 (0%, 95% CI 0–0.48)

**Table 7:** Allergen-specific metrics between detected sensitization and doctor-diagnosed food allergy for individual allergens among schoolchildren; N = 793. ∞—infinite OR.

Allergen	McNemar’s *p*-Value	Cohen’s Kappa (κ)	Odds Ratio	Odds Ratio, Lower	Odds Ratio, Upper	Sensitivity	Specificity
Hen’s egg	9 × 10^−8^	0.087	20.650	1.459	291.342	0.053	0.997
Cow milk	0.0013	0.089	22.338	0.364	447.276	0.056	0.997
Wheat flour	0.0614	−0.006	0.000	0.000	183.418	0.000	0.996
Soybean	0.0133	0.198	∞	2.234	∞	0.111	1.000
Peanut	0.0005	0.123	∞	1.330	∞	0.067	1.000
Corn flour	0.0736	0.000	0.000	0.000	∞	0.000	1.000
Fish	0.0244	0.104	19.556	0.352	262.942	0.071	0.996
Nuts	4 × 10^−10^	0.044	∞	0.458	∞	0.024	1.000
Rice	1	0.000	0.000	0.000	∞	0.000	1.000

**Table 8:** Multivariable logistic regression analysis results for total sensitization rate, according to age, sex and region of participant origin. CI—confidence interval.

Variable	OR	2.5% CI	97.5% CI	*p*-Value
Intercept	0.6043152	0.4684428	0.7773827	0.0001
Age category: school-aged children	1.7446280	1.4242357	2.1412162	0.0000
Sex: female	0.7181398	0.5958965	0.8650148	0.0005
Region: Slavonia	0.8083997	0.6329680	1.0330569	0.089
Region: Dalmatia	1.1324473	0.8765702	1.4650864	0.342

**Table 9:** Multivariable logistic regression analysis results for total polysensitization rate, according to sex, age and region of participant origin. CI—confidence interval.

Variable	OR	2.5% CI	97.5% CI	*p*-Value
Intercept	0.3561173	0.2679120	0.4701297	0.0000
Age category: school-aged children	1.9079455	1.5147104	2.4117458	0.0000
Sex: female	0.6191807	0.5019607	0.7630417	0.0000
Region: Slavonia	0.6470926	0.4933604	0.8501839	0.002
Region: Dalmatia	1.0254305	0.7752539	1.3599320	0.861

**Table 10:** Multivariable logistic regression analysis results for sensitization rates to food allergens, according to sex, age and region of participant origin. CI—confidence interval.

Variable	Odds Ratio	2.5% CI	97.5% CI	*p*-Value
Intercept	0.2498182	0.1774572	0.3469704	0.0000
Age category: school-aged children	0.9428684	0.7048424	1.2642432	0.693
Sex: female	0.7503873	0.5707483	0.9857670	0.039
Region: Slavonia	0.5857307	0.4169943	0.8252617	0.002
Region: Dalmatia	0.6455937	0.4535062	0.9207840	0.015

**Table 11:** Multivariable logistic regression analysis results for polysensitization rates to food allergens, according to sex, age and region of participant origin. CI—confidence interval.

Variable	Odds Ratio	2.5% CI	97.5% CI	*p*-Value
Intercept	0.09371229	0.05652202	0.1494589	0.0000
Age category: school-aged children	1.19608547	0.78744810	1.8306874	0.404
Sex: female	0.52387179	0.34948327	0.7776325	0.001
Region: Slavonia	0.59768858	0.36136044	0.9972578	0.046
Region: Dalmatia	0.88479086	0.53869351	1.4719895	0.632

## Data Availability

The data presented in this study are available upon request from the corresponding author, under specific conditions. The data are not publicly available due to ethical restrictions (sensitive data).

## Supplementary Materials

The following supporting information can be downloaded at https://www.mdpi.com/article/10.3390/children13020234/s1. Figure S1. Ethics committee approvals for the IMPTOX study in Croatian; Figure S2. Ethics committee approvals for the IMPTOX study—translation to English; Figure S3. Ethics committee approvals for the P4 study in Croatian; Figure S4. Ethics committee approvals for the P4 study—translation to English; Table S1. Full list of allergens used in skin prick testing of participants; Table S2. Questionnaire on lifestyle and dietary habits regarding risk for exposure to MNPs.

## Abbreviations

The following abbreviations were used in this manuscript:

CI	Confidence interval
EAACI	European Academy of Allergy and Clinical Immunology
FA	Food allergy
FFQ	Food frequency questionnaire
ISAAC	International Study of Asthma and Allergies in Childhood
MNPs	Micro- and nanoplastics
OR	Odds ratio
SPT	Skin prick test
