# Supplementary material for: Age-, Sex- and Region-Specific Patterns in Sensitization Rates to Food Allergens and Food Allergy Prevalence in Croatian Children: The H2020 IMPTOX and ERDF P4 Study Findings
Source: Children (Basel). 2026 Feb 6;13(2):234. doi: 10.3390/children13020234 (PMC12939351; doi:10.3390/children13020234)
Supplement: Supplementary file 1 [file children-13-00234-s001.zip › children-4101470-supplementary.pdf]

SUPPLEMENTARY MATERIALS

Age, sex and region- specific differences in sensitization rates to food allergens and food allergy prevalence in Croatian children: the H2020 IMPTOX and ERDF P4 study findings

Jan Pantlik[1], Marcel Lipej[2], Ivana Banić[1,3]*, Maja Šutić[1], Sandra Mijač[1], Petra Anić[1], Ana-Marija Genc[1], Ana Vukić[1], Antonija Piškor[1], Adrijana Miletić Gospić[4,] Željka Vlašić Lončarić[4], Milan Jurić[5], Vlatka Drinković[5], Ivana Marić[5], Mirjana Turkalj[5,6,7]*

[1] Department of Medical research, Srebrnjak Children`s Hospital, HR-10000 Zagreb, Croatia
[2] IT department, Srebrnjak Children`s Hospital, HR-10000 Zagreb, Croatia
[3] Department of Innovative diagnostics, Srebrnjak Children`s Hospital, HR-10000 Zagreb, Croatia
[4] Department of Pulmonology, Srebrnjak Children`s Hospital, HR-10000 Zagreb, Croatia
[5] Department of Allergy and Clinical Immunology, Srebrnjak Children`s Hospital, HR-10000 Zagreb, Croatia
[6] Faculty of Medicine, J.J. Strossmayer University of Osijek, HR-31000 Osijek, Croatia
[7] Faculty of Medicine, Catholic University of Croatia, HR-10000 Zagreb, Croatia
Correspondence: ibanic@bolnica-srebrnjak.hr (I.B.) and mturkalj@bolnica-srebnjak.hr (M.T.)

Figure S1. Ethics committee approvals for the IMPTOX study in Croatian. IMPTOX- Horizon 2020 IMPTOX (An innovative analytical platform to investigate the effect and toxicity of micro and nano plastics combined with environmental contaminants on the risk of allergic disease in preclinical and clinical studies, grant agreement number: 965173).

SREBRNJAK

Dječja bolnica Srebrnjak
Srebrnjak 100, Zagreb
Tel: 01 6391 100
Zagreb, 19.10.2021.
KLASA: 100-02/21-01
Ur.broj: 04-930/3-21

Članovi Etičkog povjerenstva su na 11. sjednici održanoj dana 19.10.2021. godine, s četiri (4) glasa ZA donijeli slijedeću

ODLUKU

Članak 1.

Odobrava se provođenje znanstveno istraživačkog projekta pod nazivom: EU H2020 ImpTox („ An innovative analytical platform to investigate the effect and toxicity of micro and nano plastics combined with environmental contaminants on the risk of allergic diseases in preclinical and clinical studies“), voditelja projekta izv.prof.dr.sc. Mirjane Turkalj, dr.med..

Članak 2.

Ova odluka stupa na snagu danom donošenja.

Obrazloženje

Dr.sc. Ivana Banić, mag.mol.biol. predala je Etičkom povjerenstvu Dječje bolnice Srebrnjak dana 30.09.2021.g. Zamolbu za odobrenje znanstveno istraživačkog projekta pod nazivom EU H2020 ImpTox financiranog sredstvima Europske unije za istraživanje i inovacije u sklopu okvirnog programa Obzor 2020 (Horizon 2020), Grant agreement number: 965173.
Cilj projekta je detaljnije istraživanje utjecaja mikro i nanoplastike iz okoliša, osobito u kombinaciji s drugim okolišnim onečišćivačima na ljudsko zdravlje s naglaskom na razvoj i kliničke manifestacije alergijske bolesti kod djece. Istraživanje će uključivati djecu dobi od 6 do 18 godina na nacionalnoj razini, u školama, kroz tri glavne geografske regije. Projekt je na sjednici Etičkog povjerenstva predstavio istraživač na projektu prof.dr.sc. Davor Plavec, dr.med. Članovi povjerenstva su sa 4 glasa ZA, odobrili provođenje istraživačkog projekta te je valjalo odlučiti kao u izreci Odluke.

Predsjednica Etičkog povjerenstvo
doc.dr.sc. Helena Munivrana Škvorc, dr.med.

Doc.dr.sc. Helena Munivrana Škvorc, dr.med.
specijalist pedijatar
alergolog i kl. imunolog
0785765

Dostaviti:
1. Podnositelju zamolbe
2. Arhiva

QUALITY SYSTEM CERTIFICATION
DNV·GL
ISO 9001:2015

Figure S2. Ethics committee approvals for the IMPTOX study- translation to English. IMPTOX- Horizon 2020 IMPTOX (An innovative analytical platform to investigate the effect and toxicity of micro and nano plastics combined with environmental contaminants on the risk of allergic disease in preclinical and clinical studies, grant agreement number: 965173).

SREBRNJAK

Srebrnjak Children`s Hospital
Srebrnjak 100,
HR-10000 Zagreb
Croatia
Tel: +385 1 6391 100
Zagreb, 19th October 2021

CLASS: 100-02/21-01
Rec.No.: 04-930/3-21

On 19th October 2021 at the 11th session of the Ethics Committee of Srebrnjak Children`s Hospital, with 4 votes PRO the members of the Ethics Committee have reached the following

DECISION

Article 1.

The implementation of the research project and the clinical study within the ***EU Horizon 2020 IMPTOX ("An innovative analytical platform to investigate the effect and toxicty of micro- and nano plastics combined with environmental contaminants on the risk of allergic diseases in preclinical and clinical studies")***, Principal Investigator: prof. Mirjana Turkalj, MD, PhD has been APPROVED hereby.

Article 2.

This decision becomes final on the day it was reached.

Elaboration

On 30th September 2021, Ivana Banić, PhD, MSc in Mol Biol has submitted the request for approval of the research project EU Horizon 2020 IMPTOX ("An innovative analytical platform to investigate the effect and toxicty of micro- and nano plastics combined with environmental contaminants on the risk of allergic diseases in preclinical and clinical studies"), funded by the EU Horizon framework programme for research and innovation, Grant agreement ID: 965173, along with relevant ethics related documentation.
The aim of this research project is to investigate the effects of environmental micro- and nanoplastics, especially in combination with other environmental pollutants, on human health, with a special focus on the development and clinical manifestations of allergic diseases in children. The study will involve children aged 6 to 18 years in schools nation-wide, in 3 main geographical regions in Croatia. The project research was presented by prof. Davor Plavec, MD, PhD, at this Ethics Committee session. The members of the Ethics Committee have approved the implementation of this research with 4 votes PRO, reaching the afore mentioned Decision.

The President of the Ethics Committee of the Srebrnjak Children`s Hospital
Assoc. Prof. Prim. Helena Munivrana Škvorc, MD, PhD

Deliver to:
1. The applicant
2. Archives

Figure S3. Ethics committee approvals for the P4 study in Croatian. P4- European Regional Development Fund project "The concept of personalized balanced nutrition in kindergartens using a research and development IT platform" (grant agreement number: KK.01.1.1.07.0074).

SREBRNJAK

Dječja bolnica Srebrnjak
Srebrnjak 100, Zagreb
Tel: 01 6391 100
Zagreb, 14.03.2022.
KLASA: 100-02/22-01
Ur.broj: 04-300/1-22

Članovi Etičkog povjerenstva su na 13. elektronskoj sjednici održanoj dana 14.03.2022. godine, s pet (5) glasova ZA donijeli slijedeću

ODLUKU

Članak 1.

Odobrava se provedba istraživačko-razvojnog projekta pod nazivom „*Koncept personalizirane uravnotežene prehrane u dječjim vrtićima kroz istraživačko-razvojnu informatičku platformu*", voditelj projekta izv.prof.dr.sc. Mirjana Turkalj, dr.med.

Članak 2.

Ova odluka stupa na snagu danom donošenja.

Obrazloženje

Prof.dr.sc. Davor Plavec, dr.med. podnio je Etičkom povjerenstvu dana 09.03.2022. godine Zamolbu za odobrenje provedbe istraživačko-razvojnog projekta pod nazivom „Koncept personalizirane uravnotežene prehrane u dječjim vrtićima kroz istraživačko-razvojnu informatičku platformu", KK.01.1.1.07.0074, financiranog iz Europskog fonda za regionalni razvoj, iz operativnog programa Konkurentnost i kohezija, Poziva jačanje kapaciteta za istraživanje, razvoj i inovacije. Voditelj projekta je izv.prof.dr.sc. Mirjana Turkalj, dr.med..
Članovi Etičkog povjerenstva su sa pet glasova ZA odobrili provedbu istraživanja.
Sukladno navedenom, odlučeno je kao u Izreci ove Odluke.

Predsjednica Etičkog povjerenstvo
doc.dr.sc. Helena Munivrana Škvorc, dr.med.

Doc. prim. dr. sc.
Helena Munivrana Škvorc, dr. med.
specijalist pedijatar
alergolog i klinički imunolog

Dostaviti:
1. Podnositelju zamolbe
2. Arhiva

QUALITY SYSTEM CERTIFICATION
DNV·GL
ISO 9001:2015

Figure S4. Ethics committee approvals for the P4 study- translation to English. P4- European Regional Development Fund project “The concept of personalized balanced nutrition in kindergartens using a research and development IT platform” (grant agreement number: KK.01.1.1.07.0074).

SREBRNJAK

Srebrnjak Children`s Hospital
Srebrnjak 100,
HR-10000 Zagreb
Croatia
Tel: +385 1 6391 100
Zagreb, 14th March 2022
CLASS: 100-02/22-01
Rec.No.: 04-300/1-22

On 14th March 2022 at the 13th online session of the Ethics Committee of Srebrnjak Children`s Hospital, with 5 (five) votes PRO the members of the Ethics Committee have reached the following

DECISION

Article 1.

The implementation of the research project and the clinical study within the ***EU ERDF "Concept of personalized balanced nutrition in preschool institutions using a research and development IT platform"***, Principal Investigator: prof. Mirjana Turkalj, MD, PhD has been APPROVED hereby.

Article 2.

This decision becomes final on the day it was reached.

Elaboration

On 9th March 2022, prof. Davor Plavec, MD, PhD has submitted the request for approval of the research project ERDF P4 ("Concept of personalized balanced nutrition in preschool institutions using a research and development IT platform"), funded by the EU Regional Development Fund, under the Operational Program for Competitiveness and Cohesion, which calls for capacity building for re-search, development, and innovation, Grant agreement ID: KK.01.1.1.07.0074, along with relevant ethics related documentation.

The project principal investigator is prof. Mirjana Turkalj, MD, PhD.

The members of the Ethics Committee have approved the implementation of this research with 5 votes PRO, reaching the afore mentioned Decision.

According to the aforementioned approval, this Decision has been made.

The President of the Ethics Committee of the Srebrnjak Children`s Hospital
Assoc. Prof. Prim. Helena Munivrana Škvorc, MD, PhD

Deliver to:

1. The applicant
2. Archives

Table S1. Full list of allergens used in skin prick testing of participants.

Type of allergen	Allergen species	Binomial nomenclature
House dust	House dust mite	*Dermatophagoides farinae*<br>*Dermatophagoides pteronyssinus*
Animal dander	Cat dander	*Felis domesticus*
	Dog dander	*Canis familiaris*
Grass pollen	5 grasses mix- cocksfoot, sweet vernal-grass, rye-grass, meadow grass, and timothy	*Dactylis glomerata, Anthoxanthum odoratum, Lolium perenne, Poa pratensis* and *Phleum pratense*
Weed pollen	Common ragweed	*Ambrosia elatior*
	Mugwort	*Artemisia vulgaris*
	Lichwort	*Parietaria officinalis*
Tree pollen	Common silver birch	*Betula verrucosa*
	Hazel (pollen)	*Corylus avellana*
	Olive	*Olea europaea*
	Pine	*Pinus radiata*
	Cypress	*Cupressus sempervirens*
Shrub pollen	Mimosa	*Mimosa pudica*
Mold	Alternaria	*Alternaria alternata*
	Cladosporium	*Cladosporium herbarum* or spp.
Food	Chicken hen`s egg (whole)	*Gallus spp.*
	Cow`s milk	*Bos spp.*
	Wheat flour	*Triticum aestivum*
	Corn flour	*Zea mays*
	Soy	*Glycine max (Soja hispida)*
	Peanut	*Arachis hypogaea*
	Sesame seed	*Sesamum indicum*
	Hake	*Merluccius merluccius*
	Trout	*Salmo trutta fario*
	Hazelnut	*Corylus avellana*
	Walnut	*Juglans spp.*
	Almond	*Amygdalus communis*
	Cocoa	*Theobroma cacao*
	Rice	*Oryza sativa*

Table S2. Questionnaire on lifestyle and dietary habits regarding risk for exposure to MNPs.

Question	Options (answers)
1. Have you/your child been previously diagnosed with any of the following allergic diseases?	Allergic asthma<br>Allergic rhinitis<br>Atopic dermatitis<br>Food allergy<br>None<br>All of the above

2. If you/your child have been diagnosed with one of the aforementioned allergic diseases, when was the diagnosis made?	Time in years ago
3. Are you/your child taking treatment for any of the allergic diseases mentioned in the first question?	yes
	no
	not applicable
If "YES", under "Other", enter the name of the medicine	Name of medicine
4. If you/your child have previously been diagnosed with food allergy, which food is in question?	Enter food type
5. What is the primary source of water consumed by you/your child?	Tap water
	Purchased water from a plastic bottle/container
	Water from a well
	Other (eg. rainwater)
6. If you/your child consume water or other beverages from plastic bottles (containers), how often do you do so?	every day
	several times a week
	several times a month
	I don't consume or very rarely consume bottled water/beverages
7. Do you/your child repeatedly use plastic bottles for the consumption of water or other beverages?	Yes, I reuse plastic bottles from store-bought drinks
	Yes, I use reusable plastic bottles
	I don't consume beverages in plastic bottles
8. If you/your child use plastic bottles for multiple use, do those bottles have any specific markings (eg BPA free) and if so, which ones (enter under other)?	yes
	no
	Other (state which one)
9. Do you/your child consume hot drinks and meals (coffee, tea, soup, etc.) in plastic bottles, cups or containers?	yes
	no
10. Do you/your child use objects (utensils) made of plastic materials when preparing hot drinks or meals?	yes
	no
11. Do you/your child store food or drinks in plastic containers, bags or bottles when freezing food/drinks?	yes
	no
12. Do you/your child use single-use plastic utensils (plates, spoons, forks, cups, straws, etc.) when eating food?	yes
	no
If yes, how often do you/your child do it?	daily
	sometimes

	rarely/never
13. Do you/your child drink hot tea and how often?	yes
	no
	tea bags
If you/your child consume tea, what kind of tea do you use most often?	tea in bulk (loose tea)
	not applicable
14. Do you/your child consume chewing gum and how often?	yes, daily
	yes, several times a week
	yes, several times a month
	rarely/never
	daily
15. How often do you/your child consume fish?	several times a week
	several times a month
	I/my child do not consume fish
	freshwater fish
	fresh sea fish from the Adriatic sea bought at the market
16. If you/your child consume fish, what kind of fish is it most often?	frozen commercial fish from the Adriatic sea
	frozen commercial fish from other seas
	canned fish
	yes, daily or several times a week
17. Do you/your child consume canned fish products?	yes, several times a month
	no/rarely
18. What is the most common source (type) of salt that you/your child use at home?	sea salt
	rock salt
	yes, daily/frequently
19. Do you/your child consume seafood (shellfish, crabs, octopus, cuttlefish, squid) and how often?	yes, sometimes
	yes, but rarely
	no
20. Do you avoid buying food packaged in plastic packaging?	yes
	no
21. Choose the statement that best describes your consumer habits:	when buying fruit/vegetables, I usually use plastic bags

	when buying fruit/vegetables, I usually use biodegradable bags
	when buying fruit/vegetables, I usually use paper bags
	I usually buy fruit/vegetables pre-weighed and packed in plastic packaging
	I usually buy frozen fruits/vegetables
	I most often consume fruit/vegetables grown in my own garden
22. If you grow fruit/vegetables yourself, do you use artificial fertilizers?	yes
	no
23. Do you/your child use products with microgranules (eg. face or body scrub, toothpaste with microbeads) during daily hygiene?	yes
	no
24. Do you/your child use products with glitter (eg. creams, eyeshadows, blushes)?	yes
	no
25. Do you/your child wear clothes made of artificial materials (eg. polyester or acrylic)?	yes
	no
26. How big is the settlement where you/your child live?	up to 1000 inhabitants
	up to 5000 inhabitants
	up to 50,000 inhabitants
	more than 100,000 inhabitants
27. Do you/your child live near major roads?	yes
	no
28. Do you/your child live near industrial plants?	yes
	no
